# Tuberculosis infection following administration of immune checkpoint inhibitors: a real world observational study

**DOI:** 10.3389/fphar.2025.1703831

**Published:** 2025-11-13

**Authors:** Mei Zhan, Linyan Zhou, Ran Liu, Qinran Long, Litao Huang, Ting Xu, Yan Fu

**Affiliations:** 1 Department of Pharmacy, West China Hospital, Sichuan University, Chengdu, China; 2 West China School of Pharmacy, Sichuan University, Chengdu, China; 3 Engineering Research Center of Medical Information Technology, Ministry of Education, West Hospital of Sichuan University, Chengdu, Sichuan, China; 4 Department of Pharmacy, Institute of Metabolic Diseases and Pharmacotherapy, West China Hospital, Sichuan University, Chengdu, China; 5 Department of Clinical Research Management, West Hospital, Sichuan University, Chengdu, China; 6 Division of Thoracic Tumor Multimodality Treatment, Cancer Center, West Hospital of Sichuan University, Chengdu, Sichuan, China

**Keywords:** immune checkpoint inhibitors, tuberculosis, retrospective study, immune-related adverse events, risk factors

## Abstract

**Background:**

This study aims to investigate the incidence of tuberculosis (TB) infection following administration of immune checkpoint inhibitors (ICI) and to explore the risk factors for developing TB in patients treated with ICIs.

**Research design and methods:**

We conducted a retrospective review of patients who had ICI until June 2023. Patient follow-up was extended until death or on July 2025. The primary outcome was the incidence of TB infection in patients treated with ICIs. Logistic regression was used to investigate the associations between clinical characteristics and TB infection after ICI initiation.

**Results:**

Of the 8,199 patients analyzed, 2.65% had a pre-existing TB diagnosis. The incidence of TB following ICI initiation was 1.96%, with pulmonary TB being the most frequent presentation. Logistic regression revealed that pre-existing TB (OR 3.277; [95% CI, 1.822–5.895]; p < 0.001) and male sex (OR 1.798; [95% CI, 1.173–2.756]; p = 0.007) were significantly associated with TB following ICI initiation.

**Conclusion:**

In this large, real-world cohort of cancer patients receiving ICI therapy, we observed a notable incidence of tuberculosis. These findings suggest that enhanced clinical vigilance may be warranted for these high-risk populations, and they highlight the need for prospective, controlled studies to definitively quantify the excess TB risk attributable to ICI therapy.

**Clinical Trial Registration:**

https://www.chictr.org.cn, identifier ChiCTR2300075974.

## 1 Introduction

The primary targets for checkpoint inhibition include the programmed cell death-1/programmed death-ligand 1 (PD-1/PD-L1) pathway and the cytotoxic T lymphocyte-associated antigen 4 (CTLA-4) (Wei et al., 2018). While ICI have revolutionized cancer treatment, the immune activation induced by these agents can cause immune-related adverse events (irAEs) that affect nearly all organ systems (Sharma et al., 2023). Current irAEs management guidelines recommend immediate high-dose corticosteroid therapy, with escalation to steroid-sparing immunosuppressants for refractory cases (Thompson et al., 2024; Haanen et al., 2022; Brahmer et al., 2021; Schneider et al., 2021). Notably, in rare cases, this immunostimulation may precipitate infectious complications, including tuberculosis (TB) reactivation (Anastasopoulou et al., 2019). Growing number of literature have reported the development of active TB in cancer patients following ICI therapy, thus suggesting that ICI therapy may lead to active TB disease (Picchi et al., 2018; He et al., 2023; Park et al., 2025). Zhang et al. identified significantly elevated levels of anti-PD-1 and anti-PD-L1 autoantibodies in patients with active TB, with positivity rates of 57.5% and 33.3% respectively (Zhang et al., 2025). These findings raise important clinical concerns regarding ICI therapy in TB-endemic regions. While the oncological benefits of ICIs are well-established, emerging evidence suggests they may paradoxically increase TB risk (Liu et al., 2022; Zhang et al., 2024). However, the precise incidence remains poorly characterized due to limited epidemiological data. We conducted a retrospective observational study to systematically evaluate the incidence of TB in cancer patients receiving ICI therapy and to identify the associated risk factors for TB following ICI treatment.

## 2 Methods

### 2.1 Study design and patient selection

This real-world study included patients who received at least one dose of ICI therapy at West China Hospital, Sichuan University, between 2014 and June 2023. The study aimed to constitute a comprehensive, unselected cohort; therefore, the inclusion criterion was the administration of at least one dose of an ICI, with no restrictions based on cancer type, stage, performance status, age, or concomitant treatments. We initially screened patients for possible ICI therapy from the Big Data Platform at West China Hospital, Sichuan University, based on the inclusion criteria. Eligibility and additional clinical data were confirmed through manual medical record review. Follow-up continued until death or 28 July 2025, whichever occurred first. The study was approved by the Biomedical Ethics Review Committee of West China Hospital, Sichuan University (Approval No. 2023-1064).

### 2.2 Definitions and outcomes

Patient medical records were retrospectively reviewed to extract tuberculosis diagnoses. We identified patients who had either a confirmed or suspected diagnosis of TB occurring at any time point relative to ICI initiation. To capture the full spectrum of clinical presentation, our inclusion criteria encompassed not only confirmed TB cases but also those where TB was strongly suspected based on clinical, radiological, or histological grounds. Cases were directly categorized according to the physician’s diagnostic classification, with Confirmed TB defined based on the presence of a physician’s explicit diagnosis of “confirmed” TB, and Suspected TB defined based on a physician’s diagnosis of “suspected,” “probable,” or “clinical” TB. For the analysis of TB incidence, both confirmed TB and suspected TB cases were included.

### 2.3 Data analysis and statistics

The demographic characteristics, cancer types, laboratory test results, and other relevant data were extracted from the Big Data Platform of West China Hospital, Sichuan University. Information regarding ICI therapy was retrieved from electronic medical records. Immunosuppressive agents might be a risk factor for tuberculosis reactivation (Liu et al., 2024; Zhang et al., 2023). Furthermore, glucocorticoids and other immunomodulators are commonly used in the management of irAEs. We therefore evaluated the use of immunosuppressive agents as a potential risk factor in our analysis, with a focus on medications most commonly used in the cancer patient population. Glucocorticoids were limited to systemic formulations (oral or intravenous), excluding topical or inhaled preparations. The analysis of other immunosuppressants was restricted to oral agents frequently employed in oncologic care (including mycophenolate, tacrolimus, and sirolimus). It should be noted that this definition specifically excluded certain immunosuppressive drug classes with established TB risk, such as TNF-α antagonists, as these agents are rarely used in cancer populations.

All statistical analyses were conducted using SPSS version 29.0. Continuous variables were presented as medians with interquartile ranges (IQRs) for non-normally distributed data and means ± standard deviations (SD) for normally distributed data. Categorical variables were summarized as frequencies and percentages. To identify factors associated with tuberculosis infection following ICI treatment, we performed univariate logistic regression, followed by multivariate logistic regression. Variables with a p-value <0.20 in univariate analysis were included in the multivariate model. Predictors for multivariate analysis were selected based on clinical relevance or univariate significance. Multicollinearity was assessed using variance inflation factor (VIF). Group comparisons were made using a two-tailed Student’s t-test where appropriate. Results from the multivariate analysis were reported as adjusted odds ratios (OR) with 95% confidence intervals (95% CI). A p-value <0.05 was considered statistically significant.

## 3 Results

### 3.1 Patient characteristics

In this retrospective study, we identified 8,610 patients who initiated ICI therapy between October 2014 and June 2023. After excluding 411 patients due to incomplete records (including missing ICI initiation dates), 8,199 remained for analysis. The cohort was predominantly male (74.08%), with a median age of 59.2 years (IQR 52.0–67.7), and lung cancer accounted for the largest treatment group (37.96%). Patient characteristics are shown in Table 1.

**TABLE 1:** Baseline patient demographics and clinical characteristics.

Characteristic	All	TB after ICI therapy	New onset of TB
Total, N	8199	161	148
Age, years madian [IQR]	59.2[52.0, 67.7]	59.4[52.7,68.5]	59.7[52.7, 68.7]
Sex			
Female n(%)	2125 (25.92)	27 (16.77)	25 (16.89)
Male n(%)	6074 (74.08)	134 (83.23)	123 (83.11)
Comorbidities			
Pre-existing TB n(%)	217 (2.65)	13(8.07)	0 (0.00)
Tobacco use n(%)	3296 (40.20)	65 (40.37)	57 (38.51)
Alcohol use n(%)	2399 (29.26)	49 (30.43)	43 (29.05)
The type of Immune Checkpoint Inhibitor			
PD-1 n(%)	7264 (88.60)	139 (86.34)	126 (85.14)
PD-L1 n(%)	642 (7.83)	17 (10.56)	17 (11.49)
CTLA-4 n(%)	1 (0.01)	0 (0.00)	0 (0.00)
More than one target n(%)	292 (3.56)	5 (3.11)	5 (3.38)
Primary cancer			
Lung n(%)	3112 (37.96)	64 (39.75)	60 (40.54)
Liver n(%)	801 (9.77)	9 (5.59)	9 (6.08)
Gastric n(%)	700 (8.54)	12 (7.45)	11 (7.43)
Esophageal n(%)	694 (8.46)	18 (11.18)	14 (9.46)
Multiple primary tumors n(%)	133 (1.62)	2 (1.24)	2 (1.35)
Other n(%)	2759 (33.65)	56 (34.78)	52 (35.14)

Abbreviations: TB, tuberculosis; ICI, immune checkpoint inhibitors; IQR, interquartile ranges; PD-1, programmed cell death protein 1; PD-L1, programmed cell death ligand 1; CTLA-4, cytotoxic T-lymphocyte antigen 4.

### 3.2 Incidence of TB

Of the study population, 217 patients (2.65%) were identified with a pre-existing TB diagnosis prior to initiating ICI therapy. Following ICI administration, 161 (1.96%) cases of TB were identified. Among these, 148 (1.81%) represented new-onset TB diagnosed after treatment initiation. Pulmonary TB remained the most frequent site of infection both before and after ICI exposure. The overall distribution of tuberculosis infection sites is presented in Table 2.

**TABLE 2:** Distribution of tuberculosis infection sites in patients pre- and post-immune checkpoint inhibitor treatment.

Site of tuberculosis infection	Number of cases (Pre-ICI)	Number of cases (Post-ICI)	Number of cases (new onset of TBI)
Pulmonary tuberculosis	149	123	116
Pulmonary and extrapulmonary tuberculosis	9	6	4
Lymphatic tuberculosis	13	6	5
Extrapulmonary tuberculosis (non-lymphatic)	35	19	17
Tuberculosis of unspecified site	11	7	6
Total	217	161	148

Abbreviations: ICI: immune checkpoint inhibitors.

### 3.3 Factors associated with TB after ICI therapy

Table 3 shows the results of the logistic regression analyses investigating risk factors for TB after ICI therapy. Based on univariate logistic regression, variables including sex and preexisting TB met the inclusion criterion of p < 0.2 and were considered for multivariate analysis. A collinearity diagnosis was performed, and the VIF for both variables (sex and preexisting TB) was 1.000, indicating no multicollinearity. In the multivariate logistic regression, preexisting TB (OR 3.277; [95% CI, 1.822–5.895]; p < 0.001) and male sex (OR 1.798; [95% CI, 1.173–2.756]; p = 0.007) were significantly associated with TB following ICI initiation. Other factors, including age, smoking, alcohol use, the use of immunosuppressive agents, the type of ICI and primary cancer, did not show statistically significant associations in the multivariate model.

**TABLE 3:** Logistic regression analysis of risk factors for TB following ICI therapy.

Characteristic	Univariable logistic regression				Multivariable logistic regression			
	P	OR	95%CI		P	OR	95%CI	
			Lower limit	Upper limit			Lower limit	Upper limit
Pre-existing TB	<0.001	3.373	1.882	6.047	<0.001	3.277	1.822	5.895
Age	0.520	1.004	0.991	1.017				
Sex	0.008	1.753	1.156	2.658	0.007	1.798	1.173	2.756
Tobacco use	0.839	0.968	0.704	1.330				
Alcohol use	0.895	1.023	0.729	1.436				
The use of immunosuppressive agents	0.629	0.915	0.640	1.310				
The type of immune checkpoint inhibitor								
PD-1	0.629							
PD-L1	0.202	1.394	0.837	2.322				
CTLA-4	1.000	0.000	0.000					
More than one target	0.805	0.893	0.363	2.196				
Primary cancer								
Other	0.439				0.444			
Lung	0.942	1.013	0.705	1.456	0.483	0.876	0.605	1.269
Liver	0.097	0.548	0.270	1.114	0.057	0.501	0.246	1.021
Gastric	0.592	0.842	0.449	1.579	0.558	0.828	0.441	1.555
Esophageal	0.360	1.285	0.751	2.200	0.681	1.121	0.650	1.934
Multiple primary tumors	0.674	0.737	0.178	3.053	0.589	0.675	0.163	2.805

Abbreviations: ICI: immune checkpoint inhibitors; TB: tuberculosis; PD-1: programmed cell death protein 1; PD-L1: programmed cell death ligand 1; CTLA-4: cytotoxic T-lymphocyte antigen 4.

## 4 Discussion

TB persists as a major global public health challenge and remains the leading cause of mortality among infectious diseases worldwide (Xie et al., 2025; GBD 2021 Diseases and Injuries Collaborators, 2024). China is one of the countries with a high global burden of tuberculosis. According to the World Health Organization (WHO) report, the estimated number of new tuberculosis cases in China in 2023 was approximately 748,000, with an incidence rate of 52 per 100,000 population, ranking third globally in terms of case numbers (World Health Organization, 2024). Notably, cancer patients inherently carry an increased risk of TB due to factors such as malignancy-associated immunosuppression, chemotherapy, and frequent healthcare exposures (Assefa et al., 2025; Dobler et al., 2017). As PD-1 blockade becomes more globally deployed, there is growing concern that TB-related adverse events may increase among patients receiving ICI (Zaemes and Kim, 2020; Barber et al., 2019). In light of these concerns, we conducted a large-scale retrospective study to evaluate the incidence and clinical characteristics of TB infection following ICI therapy. Our results showed that 2.65% had a pre-existing TB diagnosis prior to treatment initiation. Furthermore, the incidence of newly diagnosed TB following ICI commencement was 1.96%—a rate substantially higher than that observed in the general population (e.g., 0.052% in China in 2023). This pronounced difference underscores the considerable and specific vulnerability of cancer patients treated with ICI to tuberculosis infection or reactivation. Our study revealed that pre-existing TB and male sex were significantly associated with TB following ICI initiation. The association with male sex aligns with the general TB epidemiology in China, where the disease burden is consistently higher in males (Liu et al., 2025; Lu et al., 2025). Furthermore, a history of preexisting TB was associated with a statistically significant increase in the risk of TB after ICI therapy. This finding suggests that ICI therapy may precipitate the reactivation of latent tuberculosis infection or prior, inadequately treated TB. This clinical observation is strongly corroborated by recent studies focusing on lung cancer patients, which not only confirmed a prior TB history as a key risk factor but also demonstrated that ICI treatment itself confers a significantly higher risk of TB occurrence compared to conventional platinum-based chemotherapy or tyrosine kinase inhibitors (Park et al., 2025; Che et al., 2024).

The elevated TB incidence in ICI-treated cancer patients likely arises from multiple interconnected factors: underlying immunosuppression from both malignancy and anticancer treatments (Assefa et al., 2025); the disruption of immunological balance by ICIs, which may precipitate the reactivation of latent TB infection (Ruiz-de la Cruz and Salinas-Carmona, 2024; Suliman et al., 2021); and increased diagnostic scrutiny that may improve detection. These findings highlight the necessity of systematic TB screening and latent TB infection assessment before initiating ICI therapy, especially in high-burden regions, and underscore the need for future research on optimized prophylactic strategies. Despite these important implications, our study has several limitations. Firstly, our study has inherent limitations due to its retrospective design. Specifically, the identification of TB cases based on physician documentation in medical records introduces the potential for diagnostic heterogeneity and misclassification, as a standardized protocol was not applied. This limitation is compounded by the inability to definitively distinguish between new-onset tuberculosis and reactivation of latent infection, a nuance that is critical for mechanistic interpretation but difficult to ascertain from retrospective records. Furthermore, the lack of a control group fundamentally limits our ability to quantify the excess risk of TB attributable to ICI therapy. Secondly, as the elevated TB risk was identified primarily in retrospective analyses and case series, the findings are subject to inherent selection and reporting biases. The lack of systematic baseline latent TB infection screening across all studied cohorts limits our ability to definitively ascertain the true incidence of reactivation versus new infection. Moreover, the assessment of immunosuppressive medications was constrained by its reliance on in-hospital records, which precluded the systematic capture of all relevant agents, complete treatment histories, and detailed data on dosage and duration. Finally, the generalizability of our findings may be constrained by the overrepresentation of data from high-TB-burden regions, and the risk could differ in low-incidence settings.

Future studies should prioritize prospective cohorts with universal baseline screening to accurately quantify TB risk and establish evidence-based prophylactic guidelines. Further research is also needed to elucidate the specific immune mechanisms driving TB reactivation in ICI-treated patients.

## Data Availability

The raw data supporting the conclusions of this article will be made available by the authors, without undue reservation.
